# Scoping review of trials evaluating adhesive strategies in pediatric dentistry: where do simplified strategies lie?

**DOI:** 10.1186/s12903-021-01395-5

**Published:** 2021-01-19

**Authors:** António H. S. Delgado, Hasan Jamal, Anne Young, Paul Ashley

**Affiliations:** 1 Department of Biomaterials and Tissue Engineering, Royal Free Hospital, UCL Medical School, UCL Eastman Dental Institute, Rowland Hill Street, Hampstead, London, NW3 2PF UK; 2 Unit of Paediatric Dentistry, Department of Craniofacial Growth and Development, UCL Eastman Dental Institute, London, UK

**Keywords:** Adhesive, Children, Dental adhesive, Evidence-based dentistry, Restorative dentistry, Pediatric dentistry

## Abstract

**Background:**

Adhesive restorations allow a conservative approach to caries management and are increasingly used as a restorative option in pediatric dentistry. Placement can be difficult in children because of the cooperation required for multiple bonding steps. Due to this, it is vital to assess if novel, simpler strategies have been featured in clinical trials and if clinical trials are researching the different existing adhesive strategies.

**Methods:**

This review followed Preferred Reporting Items for Systematic Reviews and Meta-analysis adapted for Scoping Reviews (PRISMA-ScR) guidelines. PubMed/Medline, Cochrane Central, Scopus and EMBASE were used for systematic search, using free keywords and controlled search terms. Clinical trials of children requiring a restorative intervention which featured adhesive strategies were included. Only peer-reviewed trials of primary teeth restored with resin composites, published in the last 10-year period were eligible. Data charting was accomplished independently by two reviewers, and studies were summarized according to their date, type, intervention, sample size, observation period, outcomes and conclusions. Quality assessment was performed using Cochrane’s Risk of Bias 2.0 tool.

**Results:**

700 potentially relevant references were found, which after a rigorous inclusion scheme, resulted in a total of 8 eligible clinical trials. Out of these, 7 were randomized clinical trials. Most trials featured a split-mouth design and the observation period ranged from 12 to 36 months. The trials evaluated interventions of two self-adhesive composites, two bulk-fill composites, two novel composites, one compomer and eight adhesives from different strategies. Most studies (4/8) included were judged to raise some concerns regarding risk of bias, while two were classified as high risk and two as low.

**Conclusion:**

Few studies comparing adhesive strategies were found, especially adhesives in sound substrates. The existing studies do not reflect all current approaches that could be used in pediatric dentistry. Further studies addressing bioactive composites and contemporary adhesives are necessary.

## Background

Past trends have been naturally replaced by novel concepts, specifically when it comes to minimally invasive and non-invasive tendencies in dentistry [1, 2]. Cavity preparation design has changed dramatically ever since the concept of surface treatments was introduced [3]. This has allowed the implementation of adhesive protocols, paving the way for conservative restorations. Today, adhesive restorations dominate the field and continue to evolve [4], since this latter method permits the preservation of sound tooth structure while trying to emulate lost tooth tissues [5, 6].

Adhesive restorations allow a conservative approach to caries removal [7, 8], as a result they are increasingly used in pediatric dentistry. With the Minamata agreement in place, amalgam has been phased out and will no longer be used as a dental restorative material [9, 10]. Other popular restorative materials such as glass-ionomer cements/resin-modified glass ionomer cements (GMIC/RMGICs) do not have indication for all clinical scenarios as they have poor mechanical properties and limited longevity [11, 12].

Resin composites are the main alternative to amalgam but have some drawbacks. Requirements such as correct isolation and multiple step techniques must be met if clinical longevity is desired [13]. In the pediatric population, these requirements are often difficult to fulfil, since cooperation is limited [14]. Considering the recent advances in dental adhesives, contemporary strategies feature less clinical steps and adhesive strategies which are not as technique sensitive or difficult as their predecessors. These materials are adhesive systems that are based on a one-step self-etching strategy, universal bonding agents, which can be used in a multi-mode approach (with arbitrary adhesive strategy choice) and even new resin composites which have self-adhering properties and surpass the need for an adhesive all together. Current research has been focusing on these latest materials [15–17]. Since these novel adhesive strategies display simpler techniques, their use is highly recommended in the pediatric setting. To reduce the burden of oral health disease, and specifically caries, it is crucial to find an effective and non-sensitive, simple technique, also at a low cost [18].

A restorative procedure with less and simpler steps not only reduces chair time but also diminishes the chances of error in these multiple steps which are technique sensitive [19]. These can be—sufficient etching of the substrate, correct evaporation of the solvent in primers or single-bottle adhesives, while keeping moisture, proper handling of collagen in dentine or multiple layering of the composite to avoid stresses resulting from the polymerization reaction [20, 21]. In a pediatric setting there is less time to think about such events, and for this reason it is very important for current trials to look into new and easier techniques.

Clinical trials that researched adhesive protocols and simplified restorative strategies in children are clinically relevant, since they may provide evidence-based clinical guidance towards the use of certain adhesives or techniques in the pediatric population [22]. This scoping review approach intends to investigate clinical trials which focused on comparing different adhesive restorative materials in primary teeth. This will allow us to find out which types of trials are being conducted (randomized vs. non-randomized), which adhesive materials are being featured in the most recent trials, if novel strategies are being implemented or left out and if clinical differences between the materials are being found. It will also help to map the methodological flaws within the trials and the gaps in the current evidence.

## Methods

### Information sources, search strategy and eligibility

This scoping review was done in accordance with the Preferred Reporting Item for Systematic Reviews and Meta-Analysis extension for scoping reviews guidelines (PRISMA-ScR) [23]. An electronic search, from the period of July until October 2020 (last search 23rd October 2020), was conducted on the following databases: PubMed/Medline, Cochrane Central Register of Controlled Trials and Scopus for clinical studies and clinical trials using a search strategy, that followed the following format (example for PubMed): (dental bonding OR adhes* OR composite OR restoration OR resin) AND (children OR pediatric OR paediatric OR primary OR deciduous) AND (clinical OR trial OR RCT OR controlled study) AND (FDI OR USPHS). The retrieved articles were additionally hand searched for other potentially relevant articles. Trial registrations were also consulted (Clinicaltrials.gov). Where full-text articles could not be retrieved online, authors were contacted via ResearchGate (researchgate.net). The search was not restricted to language. The publication date was restricted to articles published between a 10-year time frame from July 2010 to July 2020, as the main aim was to study materials being currently used in clinical practice.

The study followed a PCC question format where: (Population) were children, (Concept) were restorative treatments using adhesive strategies and (Context) were interventive clinical trials using FDI or USPHS outcome criteria.

**Fig. 1:**
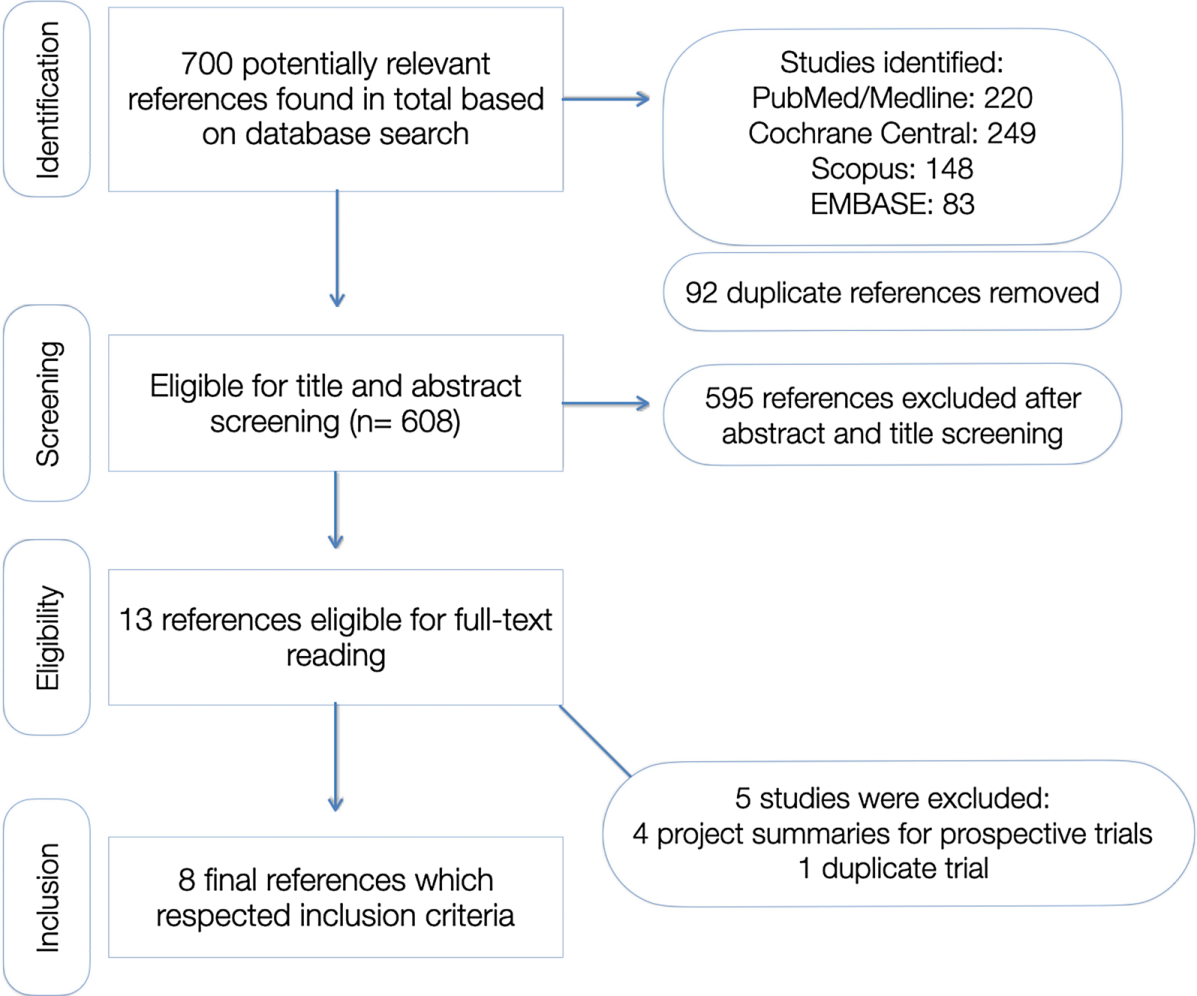
PRISMA statement flowchart followed for the scoping review

Two reviewers (A.D and H.J.) carefully screened the papers, working independently according to the inclusion and exclusion criteria. Conflicts of opinion were resolved through consensus by consulting a third reviewer (P.A.). Mendeley Desktop (v. 1.19.4) tools were used for reference organization and sorting. The workflow followed the PRISMA-ScR statement flowchart, as can be seen in Fig. 1.

### Inclusion and exclusion criteria

The inclusion criteria established for the screening of studies were as follows:

- Randomized or non-randomized clinical trials
- Participants with ages compatible with primary or mixed dentition
- Intervention studied was a comparison between bonded resin-based composites which varied the resin-based composite, underlying adhesive strategy or the application strategy/mode (i.e. self-adhesive composites)
- The restored teeth evaluated were primary teeth

Considering the exclusion criteria, these were:

- Studies evaluating indirect restorations
- Studies which evaluated a sandwich technique restoration (liner/base before final restorative material)
- Studies evaluating resin-modified glass ionomer cements (RMGICs), glass ionomer cements (GICs) or compomers
- Studies evaluating fissure sealants
- Types of excavation techniques and atraumatic restorative treatment (ART), in order to minimize bias arising from comparing studies with partial caries removal and cavity preparation techniques
- Pulp therapy or endodontic treatments
- Permanent teeth, which includes teeth affected by molar-incisor hypomineralization (MIH)

Any trials conducted partially on adults were also excluded. Reasons for exclusion of studies following full-text reading were recorded.

### Data charting and synthesis of results

A form for data charting was constructed, with relevant data entries approved in consensus by all reviewers. From each record, the following items were taken: publication author/date, study country, study design, intervention, trial conduct, study sample, sample size, follow-up period, outcomes, and conclusions. Each were extracted independently. This was done by both reviewers using a Microsoft Excel spreadsheet (A.D. and H.J.). Disagreements were solved through discussion of both reviewers. The evidence is presented in a narrative qualitative format considering differences among the studies for the interventions and materials tested, sample size, observation period and outcomes, and is also summarized in the results section.

### Quality assessment

To assess the quality of the included trials, the risk of bias was measured using the updated Cochrane Collaboration Risk of Bias Assessment Tool (RoB 2) [24]. This step is optional in scoping reviews; however, it may be considered in certain studies [23]. In this review, it is pertinent to assess risk of bias, as the individual studies included were clinical trials and their internal validity should be evaluated. Furthermore, methodological quality of the clinical trials will answer subquestions posed in this review. The overall risk of bias in the studies was classified according to three different categories—(a) low risk of bias if all categories were measured to be free of risk, (b) some concerns when one or more category raises some concerns, but not high risk in any domain or (c) high risk of bias if one or more domains are classified as high risk. Traffic light plots were made using the robvis tool [25].

## Results

### Study selection and inclusion

A total of 700 references were found in both databases, after which 608 remained when duplicates were removed (92 duplicates were found due to papers overlapping in different databases). The exclusion after the title and abstract screening lead to 13 eligible references. Of these, 5 references were clinical trials which had not been conducted or were in duplicate. The remaining 8 eligible clinical studies were included in this scoping review (Fig. 1). Seven studies were randomized clinical trials (RCT) and one of the studies was a non-randomized trial. The studies retrieved and their characteristics of the data charting conducted, are summarized in Table 1.

**Table 1:** Characteristics of the included studies in this review

Study	Study type	Participants	Age range	Intervention		Sample size		Observation period (months)	Criteria	Outcomes	Conclusion
Cavalheiro et al. [26]	RCT	62	5–8 year olds Mean: 7.1	1. Adper Single Bond (15 s etching) 2. Adper Single Bond 2 (7 s etching) (3M ESPE)		100 Class I restorations 1. n = 50 2. n = 50		18 months	FDI	Survival rates were as follows: 15 s etching = 78.3% 7 s etching = 92%	The etching time did not influence the outcome of the restorations, although a reduced time showed better tendency
Yazicioglu et al. [27]	RCT	31	4–9 years old Mean: 6.67	1. Vertise flow (Kerr) 2. Clearfil SE Bond and Filtek Z250 (Kuraray; 3M ESPE)		61 Class I restorations 1. n = 30 2. n = 31		12 months	Modified USPHS	No loss of retention was documented for either materials for a 1-year period	Good clinical scores for the self-adhesive material after 1-year. No differences for both materials
Gianetti et al. [28]	Clinical trial	28	6–12 years old Mean 8.5 years	1. Filtek Z250 (3M ESPE) 2. SDR Flowable (Dentsply)		56 Class II restorations 1. (n = 28) 2. (n = 28)		24months	Modified USPHS	Retention rates were not reported. However, marginal adaptation seems to be favoured in the SDR composite system	The authors recommend the use of SDR, a novel flowable resin for primary teeth due to its ease of application and results at 2 years time
Oter et al. [29]	RCT	80	Mean: 7.4 years	1. Filtek Z250 (3M ESPE) 2. Filtek Bulkfill (3M ESPE) Adhesive: Single Bond Universal (self-etch mode)		160 Class I restorations 1. (n = 80) 2. (n = 80)		12 months	Modified USPHS	All of the evaluated restorations were retained after 12 months (100% success rate)	Both materials were clinically successful after 1 year
Lenzi et al. [30]	RCT	44	5–10 years old Mean: 7.2 years	1. Scotchbond Universal (E&R) 2. Scotchbond Universal (SE) (3M ESPE)		90 Class I/II restorations 1. (n = 87) 2. (n = 88)		18 months	Modified USPHS	Survival rates were 100 percent until 6 months, 90.6% at 12 months and 81.4% at 18 months	The different strategies had not influence on the clinical behaviour after selective caries removal
Sabbagh et al. [31]	RCT	34	6–12 years old	1. Vertise Flow (Kerr) 2. Premise Flowable + Optibond All-In-One (self-etch)		68 Class I restorations 1. (n = 34) 2. (n = 34)		24 months	Modified USPHS	No significant difference of outcomes was found between Vertise Flow and Premise Flowable At a 2-year re-call, 3 VF restorations were lost, and 1 PF restoration	Vertise Flow showed a similar clinical behaviour to Premise Flowable at a 2-year observation period
Atabek et al. [32]	RCT	30	7–16 years old	1. Herculite Ultra (Kerr) 2. SonicFill (Kerr) Adhesive: Optibond-All-In-One (Kerr)		60 Class I restorations 1. (n = 30) 2. (n = 30)		24 months	Modified USPHS	Both intervention groups resulted in 100% retention, anatomical form and secondary caries categories	Both materials demonstrated similar clinical behaviour results at 2 years. The easier placement technique of sonic fill may be of benefit in children
Donmez et al. [33]	RCT	32	4–7 years old Mean: 5.96	1. Optibond FL (Kerr) 2. XP Bond (Dentsply) 3: AdheSE (Ivoclar) 4: G-bond (GC Corporation)		128 Class II restorations 1. (n = 32) 2. (n = 32) 3: (n = 32) 4: (n = 32)		36 months	FDI	The failure rates of the 4 groups were as follows, at 36 months: G1: 3.8% G2: 4.2% G3: 7.4% G4: 7.7%	There were no significant differences in retention rate of the different adhesives, but there were marginal adaptation differences, with E&R systems outperforming SE systems

### Interventions and materials

In all of the studies a split mouth design is mentioned, except for Cavalheiro et al. [26] in which there is no mention of paired restorations. Considering the interventions studied: five trials looked at different resin composites [27–29, 31, 32], two looked at different adhesive systems [30, 33], while one researched surface pre-treatments [26]. Cavalheiro et al. [26] study evaluated reducing the etching time of an etch-and-rinse contemporary adhesive [26]. Two studies investigated novel self-adhesive composites in comparison to traditional materials. Both studied Vertise Flow (Kerr) and compared it to traditional composites bonded with an adhesive [27, 31]. Lenzi et al. [30] evaluated two different application modes of a universal adhesive (Scotchbond Universal, 3M ESPE). One study evaluated a bulk-fill composite—Filtek Bulkfill (3M ESPE), while another study evaluated a sonic-resin placement system in bulk (SonicFill, Kerr/Kavo). One study evaluated a novel base composite, which is also placed in bulk—Smart Dentin Replacement (SDR, Dentsply) [28, 29, 32]. Only one study looked at different contemporary adhesives belonging either to an etch-and-rinse or a self-etch strategy and their performance in sound substrates—Optibond FL (Kerr), XP Bond (Dentsply), AdheSE (Ivoclar) and G-bond (GC Corporation) [33].

All of the included studies were conducted in an academic setting. Four of the RCTs were from Turkey, two from Brazil and one from Lebanon and the non-randomized trial was from Italy.

Considering operative isolation, rubber dam placement was mentioned in the six trials that evaluated either different adhesives or self-adhesive composites. Sabbagh et al. [31] went further and compared relative isolation with cotton rolls to rubber dam and found no difference.

Selective caries removal was performed in the study of Lenzi et al. [30], and this may affect the outcome assessment, putting restorations at higher risk of failure. This study passed the inclusion criteria, even though it studies selective caries removal, as it is the only one to assess an adhesive in two different strategies.

### Sample size

In this review, a total of 341 participants were included. In these patients, 723 restorations were performed. The mean number of participants in the clinical studies included was 42.6 (± 18.8) participants, with the lowest number participants being in the non-randomized trial which included only 28 participants [28]. Out of all the studies only Yazicioglu et al. [27], Oter et al. [29] and Lenzi et al. [30] reported sample and power calculations.

### Observation period

The follow-up periods for the clinical trials included in this review ranged from 12 to 36-months. One study used a 36-month follow-up period (12.5%), three studies (37.5%) used a 24-month follow-up, two studies used an 18-month period (25%) and two studies (25%) used a 12-month period.

### Outcomes

All of the studies, except Giannetti et al. [28] mentioned blinding and calibration of the examiner who assessed the outcome of the restorations. In three studies, a cross-evaluation was performed by two independent examiners. The method of calibration was mentioned in Lenzi et al. [30]. Only two studies reported the use of FDI criteria to evaluate success outcomes for the materials tested in the trials. Survival rates were 100% in three studies. In these three studies, two had a 12-month follow-up period and one had 24-months of follow-up. These studies featured self-adhesive composite Vertise Flow versus Filtek Z250/Clearfil SE Bond, Herculite Ultra/Optibond All-In-One versus SonicFill/Optibond All-In-One and Filtek Z250/SingleBond versus Filtek Bulkfill/SingleBond.

None of the 8 trials included found differences between the materials that were tested, and all materials used were deemed clinically acceptable in children.

### Quality assessment: risk of bias

**Fig. 2:**
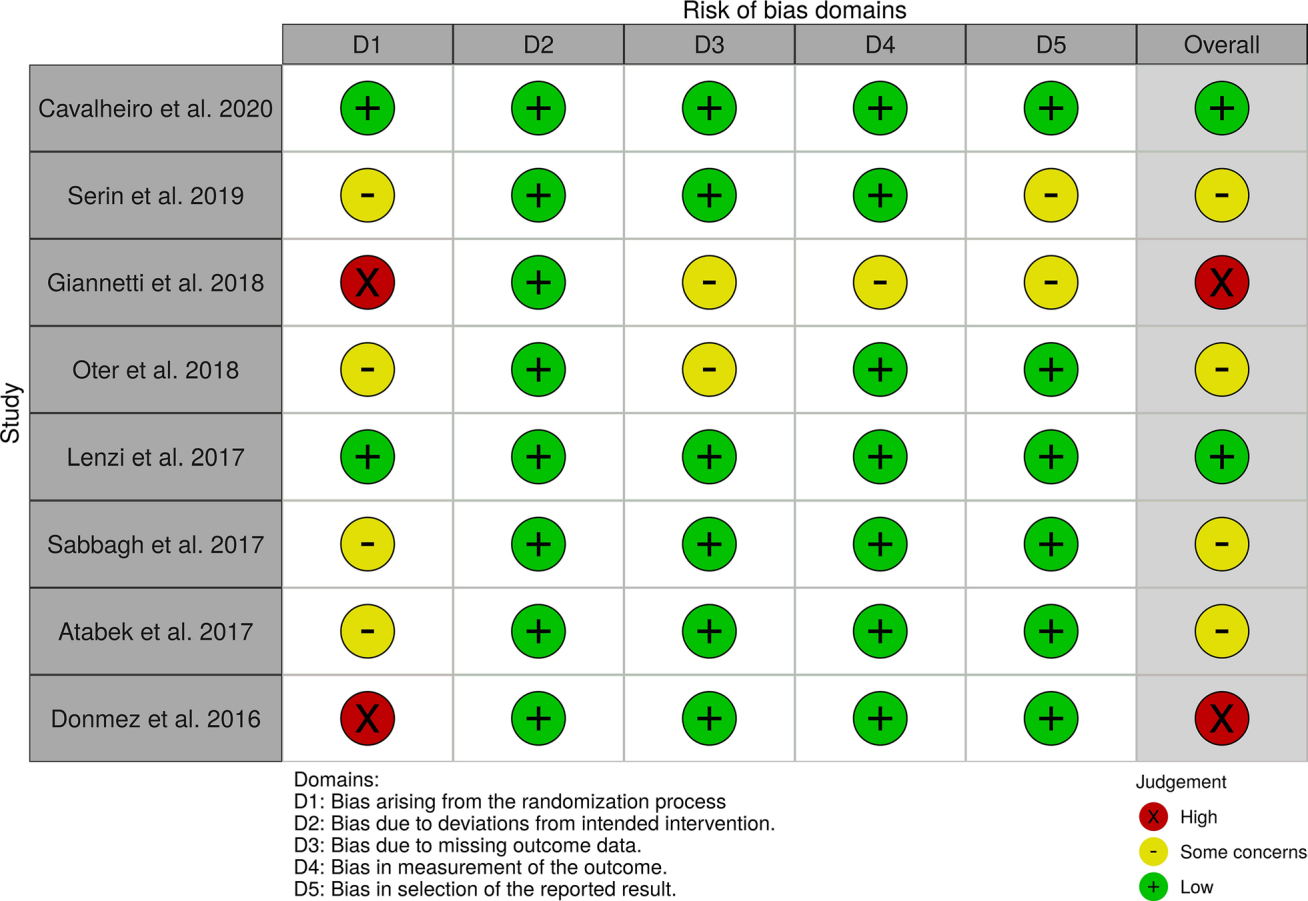
Quality assessment adapted from Cochrane’s Collaboration Tool (RoB2) for randomized controlled trials

Overall, most studies were classified as raising some concerns (4/8–50%) [27, 29, 31, 32], while 2 studies were judged to be high-risk bias [28, 33], and two were classified as low-risk bias [26, 30]. The results for each domain and the overall judgment are illustrated in Fig. 2.

## Discussion

The aim of this study was to identify and provide an updated descriptive analysis of the studies which evaluate different adhesive strategies investigated, or lacking, in pediatric dentistry.

The American Academy of Pediatric Dentistry (AAPD), in its guideline for restorative treatment, recognizes strong evidence, derived from RCTs, systematic reviews or meta-analysis, for composite use in class I or class II restorations of primary teeth. Evidence in favor for treatment of class V, based on lower evidence clinical trials was also found, and composite restorations were confirmed to have more substantiated evidence than any other material, in primary teeth [34]. Nonetheless, reducing current clinical steps without compromising quality is required.

Recent evidence suggests the failure rate seen in restorative treatments of primary teeth may be linked to the children’s behaviour during placement. If simpler and less time-consuming techniques are used, in a controlled environment, less variation in the failure rate of composites will be seen [35]. Simplification of the existing adhesive techniques would involve the reduction of steps in adhesive systems, with preference towards single-bottle and single application strategies, or eliminating the need for an adhesive altogether, by using self-adhesive restorative materials. Using bulk-fill resin composites in a single application step would also reduce chair time and avoid unnecessary layering techniques and several polymerisation cycles associated to traditional incremental-fill [36]. Contemporary flowable composites which allow less control by the clinician, to achieve adaptation in difficult access areas, facilitating technique and placement, should also be featured in trials. These may be self-adhesive or not [37, 38].

Considering the RCTs found, Lenzi et al. [30] and Donmez et al. [33] both tested the clinical application of bonding agents but portrayed different intervention types [31]. Donmez et al. [33] designed a study to assess four different commercial adhesives. Even though four different adhesives were tested, this research team did not include recent strategies such as universal adhesives which benefit from chemical adhesion, with the introduction of functional monomers alike 10-methacryloxydecyl dihydrogen phosphate (10-MDP) [39]. These monomers have affinity for the calcium in the hydroxyapatite, the mineral apatite present in mineralized tooth structures [40, 41]. Due to chemical adhesion they are classified as multimode adhesives, with the possibility of using them in three possible adhesive strategies, including as a single-step application, if the materials are used in a self-etch adhesive strategy [17]. Since many universal bonding systems are available in the market, with different functional monomers and pH levels, this becomes an easy possibility for a material choice in pediatric appointments. As they are multimodal, such systems can be used in different adhesive strategies for adults or alternative clinical scenarios such as indirect restorations [17, 42]. Optimization of these materials lead to the development of newer universal systems, such as ones containing monomers with different hydrophilic chemistry. This has been shown to reduce application times and lessen technique sensitivity even further and may be favourable for children [43, 44].

Lenzi et al. [30] conducted a clinical study that researched two of the possible different adhesive strategies within a universal adhesive. This study evaluated Scotchbond Universal (3M, ESPE) but as a limitation, it did not compare this adhesive system to other different commercial adhesives. Furthermore, this study only provided evidence concerning two out of the three possible adhesive strategies that can be used with this product: etch-and-rinse or self-etch, as selective enamel etching was left out deliberately of the experimental design. This last adhesive strategy is considered the gold standard of contemporary practice in universal adhesives. However, this strategy may not make sense in a pediatric setting as it is very difficult to control and limit acid application to enamel only. In addition, the surface area and structure of enamel and dentine are known to be different in primary teeth [45]. No differences in success of the materials used in these trials were found, which indicates that simpler strategies can be equally as effective in children, at least for short-term results, and should possibly be favoured.

Two RCTs featuring self-adhesive flowable composite Vertise Flow (Kerr, USA) were included in this review, since this material can be considered a simplified adhesive strategy which does not advocate the separate application of an adhesive. These recent materials are being currently researched and should be considered a promising option, especially in pediatric dentistry, to overcome difficulties during procedures and to reduce total appointment time. The two trials included in this review found no differences when compared to a conventional flowable or a conventional packable composite bonded via an adhesive. Due to their lower viscosity and organic composition which includes monomers with acidic functional groups, such as GPDM (in the case of Vertise Flow), or the popular 10-MDP, they are able to chemically interact with tooth substrates [46]. With the continuous development of research in this area, further RCTs taking into account these materials should be expected in the future. Currently there are other composites in the market with self-adhesive properties, such as Constic (DMG, Germany) and SureFill One (Dentsply, Germany) [17, 47]. Considering the results of the RCTs shown in this study, these may also constitute a good alternative to be used in children, especially in cavities where retention is not absolutely necessary. Traditional flowable composites requiring adhesives should also be investigated in trials, as these also facilitate the restoration placement in children. Mechanical properties and shrinkage of these materials are frequently questionable [48], however, in primary teeth, due to shorter restorative time cycles, they may constitute a viable approach.

Modern bulk-fill composites are considered a reliable and predictable choice for use in children, with 4–5 mm of achievable curing depth. As stated above, this facilitates the restorative procedure as the material can fill cavities in a single step, avoiding a layering technique [49, 50]. Recent clinical trials comparing bulk-fill composites with materials such as reinforced glass ionomers have confirmed their clinical success over the latter [51]. These materials are as aesthetic, have less technique sensitivity than conventional resin-based composites, and have comparable or superior mechanical properties [52]. Bulk-fill materials can be considered a viable alternative to other restorative techniques, as compomers are being gradually discontinued and are currently rarely used. Current evidence shows bulk-fill composites have clinical outcomes, especially longevity, comparable to conventional composites, and have been also advocated in children [53].

Dentsply’s Smart Dentin Replacement (SDR) is a flowable composite designed to be bulk fill, up to 4 mm layers, and marketed to be used in posterior primary teeth due to ease of placement and possible reduced chair time. It is urethane dimethacrylate (UDMA) based with a 68% filler load [54]. According to reports and good results in vitro, has less polymerisation shrinkage and contraction stress [48, 55]. However, the manufacturer recommends this material to be used as a base, underneath a universal composite [54]. It does not dispense the use of an adhesive. In Giannetti et al. [28] clinical trial, the material was applied with an adhesive but did not have a composite on top, and the clinical behaviour was similar to Filtek Z250 (3M ESPE). This makes it one interesting novel approach for the pediatric population [24].

Recently, Pulpdent (USA) developed a novel claim to be ionic bioactive resin-modified glass ionomer cement (RMGIC) called ACTIVA Bioactive Restorative [56]. This composite has a pediatric version—ACTIVA Kids. It was initially marketed as a self-adhesive RMGIC, but after showing unsatisfactory results, the manufacturer now recommends its use with an adhesive [57, 58]. While performing the systematic search for this review, protocols for future trials in pediatric dentistry, for this material, were found [59]. Results for these trials are not yet available, nonetheless, if they do follow the trend seen in adults, the material is far from the innovative properties described by Pulpdent. ACTIVA restored class-II cavities, using phosphoric acid-etch pre-treatment without adhesive bonding, demonstrated substantial failure 1-year post-placement [60].

Other innovative composites such as Cention N (Ivoclar Vivadent, Germany) have also been released, with promising antibacterial and self-adhesive features [58, 61, 62]. These materials should also be investigated in clinical trials as they are well within the research question raised in this study.

Higher risk of failure is associated with restorations which are larger in size [63]. This is a crucial factor which ought to be considered, since the retrieved studies found compared different types of restored cavities (Black’s class II cavities vs. class I). An added surface increases the restoration’s failure chance by 30–40% [63]. Additionally, in regard to the follow-up period of the analysed studies, the longest period found was 3-years in the Donmez et al. [33] trial. The remaining studies had shorter follow-up periods, between 1 and 3 years. It has been proven that restoration failure due to events such as secondary caries develops, mainly, several years after the placement of the restoration. Short-term studies, specifically studies which do not have follow-up periods extending beyond 3 years supply limited information on the clinical longevity of the restorations [64]. Having said that, this factor may not be as important due to natural exfoliation of primary dentition, when that is the case.

The outcomes analysed in the clinical studies that were found varied, since two different outcome criteria during clinical assessment were used: the FDI and the modified United States Public Health Service Criteria (USPHS). The FDI criteria supersedes the modified USPHS criteria [65]. It introduces a different scale in the classification scheme and even a patient centered assessment. Due to this, the FDI criteria was advocated to be used from now on, in clinical studies such as these, however, according to previous studies, its use is not yet widespread [66]. In this review, only two of the clinical studies used the FDI criteria. The appraisal of the restoration which includes the satisfaction of functional and aesthetic parameters are necessary to evaluate the quality of the treatment making patient centered outcomes pertinent. Both criteria have been proven to be suitable in primary dentition.

A split-mouth design was used in practically every trial included. This design is helpful when a comparison of two different materials is the aim and is useful in pediatric dentistry also as the variability and random error can be significantly reduced due to the elimination of inter-subject variability [67]. This design requires specific statistical analysis and sample size calculations which are in most cases absent or faulty [68, 69].

One systematic review was found during the search, although rather than analyzing RCTs, this was a review which included only in vitro studies with bond strength tests [70]. Bonding agents are generally evaluated by means of this in vitro study model [71]. This approach provides internal validity, since these tests prove which material outranks the rest and help to define materials intended to be researched in clinical studies. Even though this type of experimental research is important to address certain questions, it is crucial to stress that subsequent clinical studies are demanded to validate these materials in a population sample. Well-designed RCTs that are able to compare different restorative materials are paramount to provide clinical recommendations [72], and further RCTs are needed to cover all materials available. The findings reported in this study highlight that there are very few trials including novel materials and simplified strategies are in fact lacking.

The burden of oral health disease in children, even in middle and high-income countries, and specifically the prevalence of caries in children is worrying [73, 74]. Taking in to account the costs associated to treatments and the toll they take in health systems, easier and cheaper materials to solve the problem would be a great advantage.

This scoping review excluded studies which evaluated atraumatic restorative techniques, which generally feature RMGIC/GIC materials. This is a limitation of this study, as these trials are very common in pediatric dentistry and many exist, however, they did not fall in the scope of this review. A typology of systematic review would be prudent to include these other trials in the future.

## Conclusion

More clinical studies comparing novel composites and contemporary, easier, quicker adhesives should be conducted in children. Only one study evaluated different adhesives in sound substrates in children. Existing studies do not reflect all current available approaches. The trials analysed in this review, while scarce, confirmed that the novel approaches such as bulk-fill resin composites, self-adhesive restoratives and adhesives have comparable performance to traditional materials.

## Data Availability

Not applicable.

## Abbreviations

10-MDP: 10-Methacryloyloxdecyl dihydrogenphosphate (monomer)
ART: Atraumatic restorative technique
GIC: Glass ionomer cement
GPDM: Glycidil-phosphate dimethacrylate (monomer)
FDI: International Dental Federation
MIH: Molar-incisor hypomineralization
PRISMA: Preferred Reporting Item for Systematic Reviews and Meta-Analysis
RCT: Randomized clinical trial
RMGIC: Resin-modified glass ionomer cement
USPHS: United States Public Health Service
